# Clinician Search Behaviors May Be Influenced by Search Engine Design

**DOI:** 10.2196/jmir.1396

**Published:** 2010-06-30

**Authors:** Annie YS Lau, Enrico Coiera, Tatjana Zrimec, Paul Compton

**Affiliations:** ^2^School of Computer Science and EngineeringUniversity of New South WalesSydneyAustralia; ^1^Centre for Health Informatics, Australian Institute of Health Innovation, University of New South Wales, AustraliaAustralian Institute of Health InnovationUniversity of New South WalesSydneyAustralia

**Keywords:** Clinician, search behavior, information retrieval, Internet

## Abstract

**Background:**

Searching the Web for documents using information retrieval systems plays an important part in clinicians’ practice of evidence-based medicine. While much research focuses on the design of methods to retrieve documents, there has been little examination of the way different search engine capabilities influence clinician search behaviors.

**Objectives:**

Previous studies have shown that use of task-based search engines allows for faster searches with no loss of decision accuracy compared with resource-based engines. We hypothesized that changes in search behaviors may explain these differences.

**Methods:**

In all, 75 clinicians (44 doctors and 31 clinical nurse consultants) were randomized to use either a resource-based or a task-based version of a clinical information retrieval system to answer questions about 8 clinical scenarios in a controlled setting in a university computer laboratory. Clinicians using the resource-based system could select 1 of 6 resources, such as PubMed; clinicians using the task-based system could select 1 of 6 clinical tasks, such as diagnosis. Clinicians in both systems could reformulate search queries. System logs unobtrusively capturing clinicians’ interactions with the systems were coded and analyzed for clinicians’ search actions and query reformulation strategies.

**Results:**

The most frequent search action of clinicians using the resource-based system was to explore a new resource with the same query, that is, these clinicians exhibited a “breadth-first” search behaviour. Of 1398 search actions, clinicians using the resource-based system conducted 401 (28.7%, 95% confidence interval [CI] 26.37-31.11) in this way. In contrast, the majority of clinicians using the task-based system exhibited a “depth-first” search behavior in which they reformulated query keywords while keeping to the same task profiles. Of 585 search actions conducted by clinicians using the task-based system, 379 (64.8%, 95% CI 60.83-68.55) were conducted in this way.

**Conclusions:**

This study provides evidence that different search engine designs are associated with different user search behaviors.

## Introduction

Searching for information on the Web to support decision making is now an important part of clinician practice [1]. While much research focuses on the design of retrieval algorithms to identify potentially relevant documents, there has been little examination of the way that different search engine capabilities influence search behavior. Yet, to develop information retrieval systems that actively support decision making, it is necessary to understand the complex process of how people search for and review information when making decisions [2] and to design search user interfaces appropriate for these needs.

Recent studies of clinical search strategies have concentrated on methods of optimizing queries sent to information retrieval systems that enhance the performance of the retrieval. Hoogendam and colleagues conducted a prospective observational study of how physicians at a hospital used PubMed to search for information during their daily clinical activities [3]. They found that the likelihood of physicians viewing article abstracts returned from PubMed increased as the number of terms contained in a search query increased. Haase and colleagues investigated the optimal performance for different search engines in retrieving clinical practice guidelines by combining different search query terms [4]. Our own prior analysis of information searching by clinicians used a Bayesian belief revision framework to retrospectively model how documents might influence decisions during and after a search session [5]; the analysis demonstrated that clinicians can experience cognitive biases while searching for online information to answer clinical questions [6].

Few studies have looked at how clinicians reformulate queries and select sources to retrieve information during a search session to answer clinical questions. In previous studies, we have shown that a task-based search engine design allows for faster clinical decision making (ie, “decision velocity”) compared with purely resource-based engines at no cost in correctness of answers [7]. Similar results with respect to search times have been noted by others for the use of topic-specific “infobuttons” [8]. In the current study, we sought to understand the basis for these performance variations, by testing whether differences in search engine interface design are associated with any differences in user search behaviors.

## Methods

### Participants and Study Design

In all, 75 clinicians (44 doctors and 31 clinical nurse consultants) practicing in the state of New South Wales, Australia, were recruited to use an online information retrieval system to answer questions on 8 clinical scenarios within 80 minutes in a controlled setting in a university computer laboratory (Table 1) [9]. Participants had an average of 17 years of clinical experience, with the majority having rated their computer skills as good to excellent and having reported use of an online information retrieval system once per week or more.

Participants were randomly allocated to use either a resource-based or a task-based version of an online information retrieval system to answer the 8 questions. All participants were given a brief written orientation tutorial regarding their allocated system. Questions were presented in random order. Each participant was asked to use the allocated system to locate documentary evidence to help answer each question. Participants were asked to work through the questions as they would in a real clinical setting and not spend more than 10 minutes on any one question.

**Table 1:** Clinical questions presented to participants [9]

Question	Expected Correct Answer
Does current evidence support the insertion of tympanostomy tubes in a child with normal hearing?	No, not indicated
What is the best delivery device for inhaled medication for a child during moderate asthma attack?	Spacer (holding chamber)
Is there evidence for the use of nicotine replacement therapy after myocardial infarction?	No, use is contraindicated
Is there evidence for increased breast and cervical cancer risk after in vitro fertilization (IVF) treatment?	No evidence of increased risk
Is there evidence for increased risk of sudden infant death syndrome (SIDS) in siblings of baby who died of SIDS?	Yes, there is an increased risk
What is (are) the anaerobic organism(s) associated with osteomyelitis in diabetes?	*Peptrostreptococcus, Bacteroides*
Does existing evidence demonstrate that glucosamine has a disease-modifying role in osteoarthritis?	Conflicting evidence
Should epinephrine be given with the antivenom to prevent anaphylaxis?	Conflicting evidence

### Resource-based System Versus Task-based System

The search systems used by participants were essentially identical in that both systems allowed users to first select a profile (ie, search filter) to delimit their search and then to enter keywords to specify the focus of their search. The resource-based system first required clinicians to select a profile by specifying one of six online resources. These included PubMed, MIMS (a pharmaceutical database), Therapeutic Guidelines (an Australian synthesized evidence source focusing on guidelines for therapy), the Merck Manual, Harrison’s Principles of Internal Medicine, and HealthInsite (a government-funded consumer-oriented health database). Of the six resources, five presented evidence in a predigested, summarized form with references available for follow-up.

**Figure 1:**
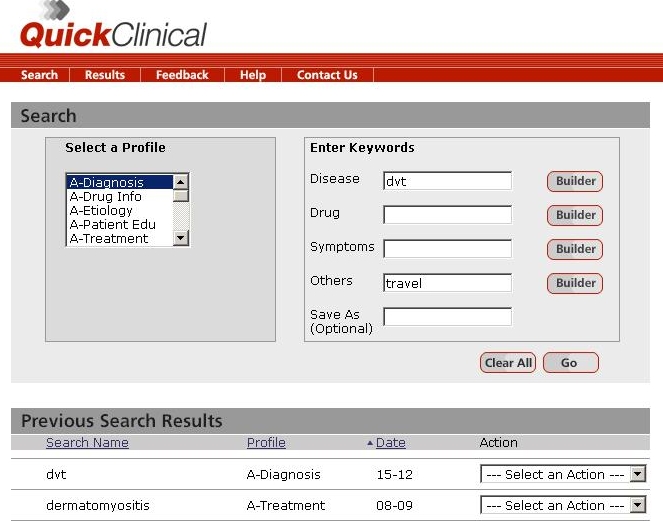
Screenshot showing Quick Clinical, the task-based query user interface

The task-based system first required the clinicians to select a profile by selecting one of six clinical tasks: diagnosis, drug information, etiology, patient education, treatment, and other (Figure 1). Four keyword categories were available for both systems: disease, drug, symptom, and other. Clinicians could enter keywords under one or more of these categories. Quick Clinical (University of New South Wales, Sydney, Australia), the task-based information retrieval system, utilized meta-search filters to simultaneously search across a set of disparate information sources [10]. This task-based system has been demonstrated to be effective and efficient in searching and delivering information in various technical, laboratory, and longitudinal evaluation studies [9-14].

### Coding of Search Actions

System logs unobtrusively capturing participants’ interactions with the systems were coded and analyzed for their search actions and query reformulation strategies. For each clinical scenario question, participants were able to reformulate queries and conduct a sequence of searches as they explored information to assist in answering the question. We first coded these query reformulations by the change in profile selection (task or resource) between consecutive searches in a session as “new profile,” “same profile,” or “previously used profile.” We next coded the keyword changes, as indicating a syntactic and/or a semantic reformulation [14]. Examples of syntactic reformulations include changing the following: the use of capitalization, the order of words, the conjunctions used between words, word spacing, or the typographic of the words (ie, variants of the base form of the word) used in the query (Table 2). Semantic reformulations include adding, removing, or replacing keywords.

**Table 2:** Examples of syntactic query reformulation

Syntactic Query Reformulation	Explanation
Change capitalization	Change capitalization of the words in a query, for example, change “IVF” to “ivf”
Change word order	Change the way words are ordered in a query, for example, “asthma diagnosis” to “diagnosis asthma”
Change conjunction	Remove, add, replace, or reorder conjunctive terms used in a query, such as “AND,” “OR,” or ”NOT”
Change spacing	Split, group, or merge words in a query by using punctuation symbols, for example, by using quotation marks to group words together to form a term or by transforming keywords, such as “heart-attack” to “heart attack”
Change typographic	Change stems, plurals, or spelling variations of words, for example, changing “run” to “running,” “apple” to “apples,” or “behavior” to “behaviour”

### Quantitative Analyses

Chi-square analyses and the test for difference between proportions were conducted to detect statistically significant differences in profile and query search actions between clinicians using the resource-based and task-based systems.

## Results

Of 75 clinicians, 39 were randomly allocated to use of the resource-based system and 36 to use of the task-based system. Two resource-based scenarios were not completed, giving a total of 310 (ie, 39×8−2) search sessions, 1708 searches, and 1455 document accesses using the resource-based system. The task-based system generated 288 (ie, 36×8) search sessions, 873 searches, and 1136 document accesses.

### Next Action in a Search Sequence

Chi-square analyses conducted of data presented in Table 3, Table 4, and Table 5 showed statistically significant differences in the next action in a search sequence between the resource-based and task-based systems. These significant differences included (1) selecting the next profile in a search sequence (χ^2^_2_ = 103.45, *P* < .001) (Table 3), (2) reformulating keywords (χ^2^_3_ = 59.37, *P* < .001) (Table 4), and (3) both selecting the next profile and reformulating keywords (χ^2^_11_ = 165.33, *P* < .001) (Table 5).

**Table 3:** Comparison of next profile actions between resource-based and task-based systems

Search System	Next Profile Action N (%) (95% CI)		
	Same Profile	Previous Profile	New Profile
Resource-based (n=1398)	557 (39.8%) (37.31 to 42.43)	202 (14.4%) (12.70 to 16.39)	639 (45.7%) (43.11 to 48.33)
Task-based (n=585)	379 (64.8%) (60.83 to 68.55)	55 (9.4%) (7.29-12.04)	151 (25.8%) (22.43-29.51)

**Table 4:** Comparison of next query reformulation actions between resource-based and task-based system users

Search System	Next Query Reformulation Action N (%) (95% CI)			
	No Change	Syntactic Change^a^	Semantic Change^b^	Syntactic and Semantic Changes
Resource-based (n=1398)	588 (42.1%) (39.50-44.67)	294 (21.0%) (18.97-23.24)	326 (23.3%) (21.18-25.61)	190 (13.6%) (11.89-15.49)
Task-based (n=585)	144 (24.6%) (21.30-28.26)	137 (23.4%) (20.17-27.02)	179 (30.6%) (27.00-34.45)	125 (21.4%) (18.24-24.87)

^a^ Syntactic change refers to changes in capitalization, typographic, ordering of words, spacing of words, and adding or removing conjunctions in a query.

^b^ Semantic change refers to adding, removing, or replacing words in a query.

The test for difference between proportions revealed that clinicians using the resource-based system were 19.5% more likely to select a new profile and apply no changes to keywords (Z =11.43, *P* < .001), and 5.9% more likely to select a profile that was previously visited and apply no changes to keywords (Z = 5.80, *P* < .001) (Table 5). Also, clinicians using the task-based system were 7.8% more likely to keep the same profile in a sequence of search actions (Z = –5.28, *P* < .001), 7.5% to keep the same profile and apply both syntactic and semantic changes to the query (Z = –4.69, *P* < .001), and 6.5% to keep the same profile and apply semantic changes to the query (Z = –3.37, *P* < .001) (Table 5). Further, task-based clinicians seldom accessed a profile that had been previously visited (9.4%, 95% CI 7.29-12.04) (Table 3).

**Table 5:** Comparison of profile and query reformulation actions between resource-based and task-based systems

Next Profile Action + Next Query Reformulation Action	Resource-based (n=1398) N (%) (95% CI)	Task-based (n=585) N (%) (95% CI)	Z	*P*
Same profile + no change	67 (4.8%) (3.79-6.04)	74 (12.6%) (10.20-15.59)	–5.28	<.001
Same profile + syntactic changes only	193 (13.8%) (12.10-15.71)	99 (16.9%) (14.10-20.18)	–1.73	.08
Same profile + semantic changes only	203 (14.5%) (12.77-16.46)	123 (21.0%) (17.92-24.51)	–3.37	<.001
Same profile + syntactic and semantic	94 (6.7%) (5.53-8.16)	83 (14.2%) (11.59-17.25)	–4.69	<.001
Previous profile + no change	120 (8.6%) (7.23-10.17)	16 (2.7%) (1.69-4.40)	5.80	<.001
Previous profile + syntactic changes only	18 (1.3%) (0.82-2.03)	10 (1.7%) (0.93-3.12)	–0.69	.49
Previous profile + semantic changes only	36 (2.6%) (1.87-3.54)	17 (2.9%) (1.82-4.60)	–0.41	.68
Previous profile + syntactic and semantic	28 (2.0%) (1.39-2.88)	12 (2.1%) (1.18-3.55)	–0.07	.94
New profile + no change	401 (28.7%) (26.37-31.11)	54 (9.2%) (7.14-11.85)	11.43	<.001
New profile + syntactic changes only	83 (5.9%) (4.81-7.30)	28 (4.8%) (3.33-6.83)	1.06	.29
New profile + semantic changes only	87 (6.2%) (5.07-7.61)	39 (6.7%) (4.91-8.98)	–0.36	.72
New profile + syntactic and semantic	68 (4.9%) (3.85-6.12)	30 (5.1%) (3.62-7.23)	–0.24	.81

### Search Actions During a Session

We examined search behaviors at the beginning, middle, and end of a search sequence. At the beginning of a search sequence, query reformulation was the most frequent choice for both systems (Table 6). In the middle of a session, clinicians using the resource-based system were 26.6% more likely to change profile only (Z = 10.21, *P* < .001) (Table 6), and clinicians using the task-based system were 20.7% more likely to reformulate query only (Z = –6.06, *P* < .001) (Table 6). At the end of a sequence, clinicians using the resource-based system were 26.7% more likely to change profile only (Z = 6.50, *P* < .001) (Table 6), and clinicians using the task-based system were 14.9% more likely to reformulate query only (Z = –2.75, *P* = .006) (Table 6).

**Table 6:** Search action between resource-based and task-based systems during a session

Search Action		Resource-based N (%) (95% CI)	Task-based N (%) (95% CI)	Z	*P*
**First**		(n=263)	(n=189)		
	No change	9 (3.4%) (1.81-6.37)	31 (16.4%) (11.80-22.34)	–4.45	<.001
	Change query only	115 (43.7%) (37.86-49.77)	100 (52.9%) (45.81-59.90)	–1.93	.05
	Change profile only	82 (31.2%) (25.89-37.01)	24 (12.7%) (8.68-18.20)	4.93	<.001
	Change query and profile	57 (21.7%) (17.12-27.04)	34 (18.0%) (13.17-24.09)	0.98	.33
**Middle**		(n=913)	(n=267)		
	No change	47 (5.1%) (3.89-6.78)	32 (12.0%) (8.62-16.43)	–3.23	.001
	Change query only	303 (33.2%) (30.21to 36.31)	144 (53.9%) (47.94-59.81)	–6.06	<.001
	Change profile only	359 (39.3%) (36.20-42.53)	34 (12.7%) (9.26-17.27)	10.21	<.001
	Change query and profile	204 (22.3%) (19.76-25.16)	57 (21.3%) (16.86-26.65)	0.35	.73
**Last**		(n=222)	(n=129)		
	No change	11 (5.0%) (2.79-8.65)	11 (8.5%) (4.83-14.62)	–1.25	.21
	Change query only	72 (32.4%) (26.62-38.84)	61 (47.3%) (38.87-55.86)	–2.75	.006
	Change profile only	80 (36.0%) (30.01-42.54)	12 (9.3%) (5.40-15.56)	6.50	<.001
	Change query and profile	59 (26.6%) (21.20-32.75)	45 (34.9%) (27.20-43.44)	–1.62	.11

### Consecutive Search Actions

Table 7 displays comparisons of the frequencies of use of consecutive pairs of actions anywhere within a sequence between the two systems. For clinicians using the resource-based system, the pair “change profile only” followed by “change profile only” was 18.6% more likely (Z = 13.88, *P* < .001) (Table 7). Among clinicians using the task-system, the pair “change query only” followed by “change query only” was used 17.8% more frequently compared with clinicians using the resource-based system (Z = –6.95, *P* < .001) (Table 7).

**Table 7:** Consecutive search actions in a session between resource-based and task-based systems

Current Search Action	Next Search Action	Resource-based (n=1135)^a^ N (%) (95% CI)	Task-based (n=396)^b^ N (%) (95% CI)	Z	*P*
No change	No change	10 (0.9%) (0.48-1.61)	7 (1.8%) (0.86-3.60)	–1.23	.22
	Change query only	17 (1.5%) (0.94-2.39)	28 (7.1%) (4.94-10.03)	–4.17	<.001
	Change profile only	20 (1.8%) (1.14-2.71)	6 (1.5%) (0.70-3.27)	0.34	.73
	Change query and profile	8 (0.7%) (0.36-1.38)	16 (4.0%) (2.50-6.46)	–3.27	.001
Change query only	No change	16 (1.4%) (0.87-2.28)	26 (6.6%) (4.52-9.45)	–3.99	.001
	Change query only	157 (13.8%) (11.95-15.96)	125 (31.6%) (27.18-36.30)	–6.95	<.001
	Change profile only	122 (10.7%) (9.08-12.69)	21 (5.3%) (3.49-7.97)	3.75	<.001
	Change query and profile	107 (9.4%) (7.86-11.27)	42 (10.6%) (7.94-14.03)	–0.66	.51
Change profile only	No change	21 (1.9%) (1.21-2.81)	5 (1.3%) (0.54-2.92)	0.85	.40
	Change query only	103 (9.1%) (7.54-10.89)	24 (6.1%) (4.11-8.86)	2.05	.04
	Change profile only	228 (20.1%) (17.86-22.52)	6 (1.5%) (0.70-3.27)	13.88	<.001
	Change query and profile	74 (6.5%) (5.23-8.11)	15 (3.8%) (2.31-6.15)	2.26	.02
Change query and profile	No change	11 (1.0%) (0.54-1.73)	5 (1.3%) (0.54-2.92)	–0.46	.65
	Change query only	98 (8.6%) (7.14-10.41)	28 (7.1%) (4.94-10.03)	1.02	.31
	Change profile only	69 (6.1%) (4.83-7.62)	13 (3.3%) (1.93-5.53)	2.45	.01
	Change query and profile	74 (6.5%) (5.23-8.11)	29 (7.3%) (5.15 to 10.32)	–0.54	.59

^a^ 263 searches were excluded because the next search action was stop searching.

^b^ 189 searches were excluded because the next search action was stop searching.

## Discussion

Clinicians using the resource-based system appeared to favor a “breadth-first” search strategy, exploring different resources with the same keywords in the query before searching in a specific resource with query reformulations. Clinicians using the task-based system were provided with results from multiple resources in each search and so appeared to favor a “depth-first” search strategy, searching in the same task profile exhaustively with different keyword reformulations in the query before moving to other profiles.

We have previously shown that changes in search engine design and interface were associated with changes in clinical decision velocity, number of search actions undertaken, and ultimate decision outcome [7]. To understand the basis for such differences, we have now looked at the type of actions undertaken by users of two different systems and the sequences of these actions. While it was the intention of the experiment to detect changes in search behavior, our present analysis extends the analytic framework of the original experiments and may thus suffer from being a post hoc explanation of the observed differences. This limitation may readily be addressed by further experiments specifically designed to test for changes in search strategy.

Further study is needed to understand how clinicians assess the results of a search and formulate the next step in their strategy. We have discussed elsewhere that the process of searching can be thought of as a conversation [15] where individuals ask questions of knowledgeable agents (eg, information retrieval systems or people) to help find answers to their questions. Thinking of the interaction with a search engine as a conversation between a human with a question and a search engine with capabilities to help find an answer may help us understand the human behaviors observed in this study.

According to Grice’s conversational maxims [15], (which were originally created to describe the “rules” for effective human conversations), an answer to a question may be inappropriate for a number of reasons. The respondent may be poorly qualified to answer the question (eg, the respondent may be an inappropriate, out of date, or otherwise misleading information source); may misunderstand the question (eg, the query may not be well expressed in terms understandable by the resource); or may reply with unhelpful or irrelevant information (eg, because of poor relevance metrics of the search algorithm). We can speculate that the search actions taken by clinicians are in response to judgments they make about the progress of their “conversation” with the information retrieval system.

One can hypothesize, when clinicians are faced with a choice of several resources with no clear indication of which is the best, they scan multiple resources to gauge the "competence" of each before committing to a detailed conversation with the resource they feel best qualified to help. In contrast, clinicians with a task-based system are simultaneously receiving answers from multiple resources and so should be able to quickly form a view of the overall capabilities of the group of resources being simultaneously searched. Not faced with concerns about the competence of the system they are interacting with, clinicians focus on improving the dialogue with the system. This is done by finding different ways to ask the same question or by changing the question focus if there has been a “misunderstanding.” As a result, this could explain why users of task-based systems conduct fewer searches and consult fewer documents [7], that is, these users may not need to credential the resources they are interacting with in the same way that users of resource-based systems appear to do.

Overall, given the clear differences in the styles of user-system dialogue demonstrated in this study, and the impact of such behavior on the clinical utility of information retrieval systems, discovering ways of optimizing the dialogue between knowledge sources and users seems a productive line of further enquiry.

## Abbreviations

IVF: in vitro fertilization
SIDS: sudden infant death syndrome
